# Host/genetic factors associated with COVID-19 call for precision medicine

**DOI:** 10.1093/pcmedi/pbaa026

**Published:** 2020-07-21

**Authors:** Alain R Thierry

**Affiliations:** Research Institute of Cancerology of Montpellier, INSERM U1194, IRCM, ICM, Montpellier University, Montpellier F-34298, France

**Keywords:** COVID-19, neutrophil extracellular traps, sequelae, genetic factors, host factors, circulating DNA

## Abstract

If the current rate of infection are to be better managed, and future waves of infection kept at bay, it is absolutely necessary that the conditions and mechanisms of exposure to Severe Acute Respiratory Syndrome-Coronavirus 2 (SARS-CoV-2) be better understood, as well as the downstream severe or lethal clinical complications. While the identification of notable comorbidities has now helped to define broad risk groups, the idiosyncratic responses of individual patients can generate unexpected clinical deterioration that is difficult to predict from initial clinical features. Thus, physicians caring for patients with COVID-19 face clinical dilemmas on a daily basis. The ability to decipher individual predispositions to SARS-CoV-2 infection or severe illness, in light of variations in host immunological and inflammatory responses, in particular as a result of genetic variations, would be of great benefit in infection management. To this end, this work associates the description of COVID-19 clinical complications, comorbidities, sequelae, and environmental and genetic factors. We also give examples of underlying genomic susceptibility to COVID-19, especially with regard to the newly reported link between the disease and the unbalanced formation of neutrophil extracellular traps. As a consequence, we propose that the host/genetic factors associated with COVID-19 call for precision medicine in its treatment. This is to our knowledge the first article describing elements towards precision medicine for patients with COVID-19.

## Introduction

In the 6 months since the first cases were reported, SARS-CoV-2 has infected over 5.5 million people in 212 countries, of whom at least   350000 have died.^1^ While overall mortality rate of COVID-19 is estimated to be about 2%, 10%–20% of individuals diagnosed with COVID-19 are hospitalized, and over 50% of patients critically ill with COVID-19 die as a result of multiple organ dysfunction and severe complications. The World Health Organization (WHO) has now set out a list of the clinical syndromes associated with SARS-CoV-2. These include both asymptomatic and uncomplicated forms of the disease, pneumonia (both non-severe and severe), the potentially fatal respiratory insufficiency known as Acute Respiratory Distress Syndrome (ARDS), acute cardiac injury, heart failure, sepsis/multiple organ failure and septic shock, secondary infection, acute kidney injury, and coagulopathy/hemorrhage (Tables 1 and 2). The complications in patients with COVID-19, then, can be said to be relatively numerous; there also appears, however, to be a notable variation in those patients’ susceptibility to severe forms of COVID-19, in comparison to infection with other pathogens (Table 1).

**Table 1.** Diversity of COVID-19 clinical features from previous studies (updated 20 April 2020).^2,3^ A greater number of asterisks refers to higher frequency of clinical features/complications in lethal cases as compared to no-lethal cases.

COVID-19 clinical features/complications	Non-survivor/survivor
Life-threatening respiratory insufficiency	***
Sepsis/multiple organ failure	***
ARDS	***
Septic shock	**
Acute cardiac injury	**
Heart failure	**
Secondary infection	**
Acute kidney injury	**
Coagulopathy/hemorrhage	**
Hypoproteinemia	*
Acidosis	*

Various reports describe the comorbidities significant to COVID-19 (Table 2). These clearly account for increases in susceptibility to severe forms of the disease, given that among non-survivors about^3^ half had hypertension; 10%–20% had diabetes, immunodeficiency, coronary heart disease, cerebrovascular disease, or chronic obstructive pulmonary disease (COPD); and 2%–10% had secondary infection, cancer, kidney or liver disease (Table 2^2,4,5–9^). When comparing severe to non-severe cases of COVID-19, the most prevalent comorbidities (in decreasing order) are: COPD, diabetes, coronary heart disease, kidney disease, hypertension, and cancer.^10^ Irrespective of COVID-19 severity, the highest prevalent comorbidity by far is associated with age and, to a lesser extent, gender (older people and males being most affected).

**Table 2.** Diversity of COVID-19 comorbidities. The % range in non-survivors was calculated from previous studies.^1–11^

COVID-19 comorbidities	% Range in non-survivors
Hypertension	>50%
Pulmonary diseases	10%–20%
Cardiovascular disease	
Cerebrovascular disease	
Immunodeficiency	
Type 1 diabetes	
Liver diseases	2%–10%
Kidney diseases	
Secondary infection	
Cancer	
Obesity	<2%
Disseminated intravascular coagulation	
Chronic inflammation disease	
Rheumatoid arthritis	
Sepsis	
Sickle cell disease	
Inflammatory bowel disease	

These data highlight the wide spectrum that exists in infected individuals, ranging from the absence of symptoms to much more severe cases. SARS-CoV-2 is inarguably distinct from previous outbreaks involving SARS-CoV and MERS-CoV by (i) very high transmission rate of SARS-CoV-2, and (ii) the large variations in disease severity among infected individuals.^11^ This raises a number of preliminary questions: How to explain this diversity? Could a particular genetic background be involved? Would it be necessary to test for predispositions in infected people at the point of diagnosis?

## Inherited factors in COVID-19

Over the last few months, numerous studies have described or suggested inherited risk factors. Nguyen *et al.*^12^ reported that genetic variability across the three major histocompatibility complex (MHC) class I genes (HLA A, B, and C) may affect susceptibility to and severity of SARS-CoV-2. Thus, carriers of the HLA-B * 46:01 variant would be particularly vulnerable to SARS-CoV-2. This had already been identified as being the case for SARS-CoV. In contrast, the HLA-B * 15:03 variant would provide some protection. According to the authors, the early identification of an individual's HLA genes (a quick, inexpensive procedure) would help to anticipate the possible severity of the infection, and to identify individuals who ought to be prioritized for vaccination. A study by Zhao *et al*.^13^ involving 2173 COVID-19 patients, associated a greater risk of COVID-19 acquisition with those of blood group A, as compared with non-A blood groups, and associated a lower risk with blood group O.

Other host factors impacting individual risks, in particular because of genetic variations, were suggested or reported. Notably, in 2017, the authors identified a gene which, when silenced by a mutation, makes rodents highly susceptible to SARS-CoV. The gene in question, *Ticam2*, codes for a helper protein in the activation of a receptor family (TLR, toll like receptor) which is called upon in the mechanisms of innate immunity, and notably in the formation of neutrophil extracellular traps (NETs). The team with De Buyzere^14^ recently demonstrated that polymorphisms of the host's angiotensin-converting enzyme 2 (ACE2), which is involved in SARS-CoV-2 cell entry and infection, may explain the epidemiology of the disease worldwide, with a possible correlation between the geographic distribution of variants and the prevalence of COVID-19. Allelic variants of the *ACE2* may influence the protein's binding to the virus and its subsequent invasion of the cell.^11,14^ The frequency of*ACE1 D* varies from one country to another, especially in Europe. The variability in the prevalence of the disease in 25 countries is explained at 38% by the frequency of the *ACE1 D* gene.^14^ Variants of the TMPRSS2 protein that is also involved in SARS-CoV-2 cell entry, were thought to be causing genetic variability. Very recently, Sagkan *et al*.^15^ demonstrated that CD147/BSG, which is a new binding receptor for the SARS-CoV-2 spike protein, may show a missense mutation (p.F275V). It should be noted that the inhibition of CD147/BSG has beneficial effects in the prevention of diabetic complications involving severe acute respiratory syndrome triggered by COVID-19.^16^ In a comparative study, Ikitimur *et al*.^17^ identified genetic factors implicated in a family cluster of SARS-CoV pneumonia with a very poor outcome. Given that hypoxic conditions, as a result of activation of the coagulation cascade and micro-thrombus formation, may potentially arise in patients with COVID-19,^18^ it is possible that hypoxia-inducible transcription factors influence susceptibility to the disease.^19^ It must also be noted that newborn screening has been promoted to avoid the increased risk as a result of inherited metabolic disorders that may cause mental retardation, premature death, or adverse outcomes in early life, where SARS-CoV-2 infection occurs.^20^ Lastly, the literature describes the possible implication of allelic variants in the *DSTN, CFL1* and *CFL2, MUC5B, TERT*, and*DPP4* genes^11^ (Table 3).

**Table 3.** COVID-19 potential host/genetic factors reported from previous studies.^12–21^ Genetic factors are represented as the genes in which genetic alteration may occur.

COVID-19 inherited factors	COVID-19 genetic factors
MHC class I genes (HLA), blood group, gender (male)	*Ticam2*
	*ACE2*
	*TMPRSS2*
	*CD147/BSG*
	*DSTN, CFL1, CFL2*
	*MUC5B, TERT, DPP4*
	locus 9q34.2
	locus 3p31.21

The scientific community is now trying to address the issues of genetic susceptibility to SARS-CoV-2 infection and COVID-19 severity by combining research efforts, using existing genetic databases.^11^ For example, A. Ganna and M. Daly of the University of Helsinki have launched the COVID-19 Host Genetics Initiative, which aims to unite the community of geneticists working on the subject internationally (https://www.covid19hg.org/). Jean-Laurent Casanova, from the Necker-Enfants Malades Hospital, Paris, and Rockefeller University, New York, is coordinating a similar project to identify variants promoting the development of particularly serious forms of COVID-19 in individuals under the age of 50 (1%–5% of patients). The governments of the UK, Iceland, and Greece, as well as Harvard and Yale Universities also have major programs with comparable aims (https://www.ukbiobank.ac.uk/2020/04/covid/; https://www.decode.com/; http://www.gsrt.gr/central.aspx?sId=119I428I1089I646I488772; https://wyss.harvard.edu; https://covidtrackerct.com).

Significant results were obtained from the Severe COVID-19 GWAS Group headed by A. Franke and T. Karlsen from genomewide association study (GWAS) of severe COVID-19 with respiratory failure.^21^ They identified gene variants in two areas of the human genome that are associated with COVID-19-related death: locus 3p31.21 being associated to the disease susceptibility gene cluster, and locus 9q34.2 that coincides with the ABO group; the latter confirming ABO blood group system involvement as a genetic factor involved in COVID-19 pathogenesis as described above. Considering the statistical significance of identification of both loci, it seems possible that next generation sequencing (NGS) of corresponding gene clusters in individuals newly diagnosed with COVID-19 might be beneficial to disease management care in stratifying patients.

## NETs and by-product role in COVID-19 pathogenesis

The function of neutrophil extracellular traps (NETs, composed of chromatin decorated with granule proteins) can be regarded as a ‘double-edged sword’. As an innate immune response, on the one hand, NET formation is an efficient strategy for neutralizing invading micro-organisms.^22–26^ On the other hand, the toxicity of NETs’ exposed by-products to endothelial cells and parenchymal tissue can have detrimental effects on the host. Indeed, it is by this process that unbalanced NET formation and neutrophil activation may contribute to numerous non-autoimmune pathologies, including cystic fibrosis, sepsis, thrombosis, severe obesity, acute lung injury associated with transfusion, and pre-eclampsia or kidney diseases. It may also participate in the pathogenesis of such autoimmune diseases as lupus, type 1 diabetes, autoimmune vasculitis, and gouty arthritis, as well as less common conditions where the small blood vessels are impacted, especially those of the kidneys, lungs, and skin.^26^

We were among the very first to highlight the link between COVID-19 and NETopathies.^27^ In a recent in-depth review, we considered the way in which NETosis dysregulation can generate a harmful amplification loop between tissue damage and inflammation, and highlighted analogous biological and physiological features in COVID-19 infection.^26^ Stated in general terms, it can be said that unbalanced NET formation and neutrophil activation result in inflammatory diseases and multi-organ damage.^28^ More specifically, NET dysregulation and COVID-19 are both associated with abnormal coagulation factors, pro-thrombotic activity, and with the cytotoxicity of endothelial and epithelial cells.^29^ The notable feature of those characteristics is that they lead to systemic vascular permeability. From a biologic perspective, their patho-physiological effects include: elevated interferon levels, C reactive protein, neutrophil concentration in lung vascularization, lactate dehydrogenases and pro-inflammatory cytokines, or the presence of circulating fibrinogen. Accordingly, uncontrolled NET formation and COVID-19 may both lead to respiratory insufficiency up to ARDS, thrombosis, sepsis, acute cardiac injury, and heart failure.^26^

Patients with viral infections such as hantavirus or HIV show elevated levels of circulating NETs, and of the circulating DNA and histones that NETs typically induce. Moreover, a role clearly is played by NETs and neutrophils in the pathologies of the simian immunodeficiency virus, the Parvovirus, the chikungunya virus, the rhinovirus, influenza, and the influenza pneumonia virus.^22–25^

Although most people with COVID-19 have mild to moderate symptoms, the disease can cause severe medical complications and lead to death. Notably, the disease presents a greater risk of evolution towards a serious form in older adults or those with pre-existing chronic health conditions. Hypertension, cardiac disease, renal disease, diabetes mellitus, pulmonary disease, or obesity were found to be the most common chronic underlying health conditions in patients with COVID-19.^5^ These conditions correlate with the pathologies resulting from NET formation dysregulation (NETopathies),^26–28^ as described above.

Taken as a whole, an analogous comparison can be made between NETs’ deleterious effects^5^ and the numerous pathophysiological and biological features of COVID-19.^26,30^ Such correlation, obviously, cannot be taken as shorthand for causation. That said, we are committed to the hypothesis that SARS-CoV-2 induces a disproportionate virus-induced NET release, and that this plays a key role in the pathogenesis of COVID-19; specifically, that unbalanced NET formation leads, in part at least, to a massive and uncontrolled inflammation process, and that it is under this amplifier loop that COVID-19 progresses.^26,27^ We hypothesize that a key role in COVID-19 pathogenesis is played by a disproportionate NET release induced by the SARS-CoV-2 virus.^28^ Furthermore, we believe that pathogenic host factors may allow the virus to elude an innate immune response, which may in turn provoke chronic NET auto-stimulation, with consequences akin to those of an autoimmune-like disease.^26,28^

There are a few preliminary results demonstrating the presence of exacerbated NET formation in patients with COVID-19,^31^ and the effect of NET inhibitor treatment.^32^ However, we and other groups started clinical studies on large patient cohorts, and we believe that investigation aiming to decipher the role of NET in COVID-19 pathophysiology would provide new options for therapy, disease management, and patient stratification, possibly through the elucidation of genetic factors.

## Possible genetic variants exacerbating NETs formation

Given that NETs are certainly involved in COVID-19, genetic factors affecting NETs may be involved in the disparity in disease severity. NETosis is a complex biological phenomenon, with various stimuli and effectors. Exhaustive listing of gene variants candidates is, therefore, impossible. That said, as an illustration of the potential influence of NETs in varying host COVID-19 susceptibility, we offer the following examples of the various stages of the phenomena: (i) as NADPH oxidase is required for generation of NETs, it may illustrate how genetic variants of NET stimuli effectors may be involved in NETosis dysregulation,^33^ as observed in COVID-19^28^; (ii) the W620 polymorphism in *PTPN22* was found to disrupt its interaction with peptidylarginine deiminase Type 4 (PAD4),^34^ thus enhancing citrullination and NETosis; (iii) evaluation of the functional effects of genetic variants, missense and nonsense SNPs, indels, and copy number variations in the gene encoding human deoxyribonuclease I (DNase1), were recently identified as having potential implications for autoimmunity as well as COVID-19 susceptibility^35^; (iv) IL-26 has been identified as a potential cargo for extracellular DNA cell entry, leading to the release of pro-inflammatory cytokines in a STING and inflammasome-dependent pathway^36^; the identification of alternative splicing for IL-26 in a species in which the gene has not been inactivated has also been reported^37^; (v) as vasculitis has been associated with exacerbated NETs formation, anti-neutrophil cytoplasmic antibody (ANCA)-associated vasculitis (AAV) may be triggered by NETs and their remnants^38^; and (vi) a single nucleotide polymorphism (SNP, rs7151526) in SERPINA1 gene leads to decreased production of alpha-1 anti-trypsin (A1AT), the main protein 3 (PR3), and elastase inhibitor.^22,39^ We are convinced that elastase is one of the critical factors for epithelial and endothelial cell toxicity, micro-thrombosis, and vasculitis, and consequently believe that research on elastase genetic variants^40^ should be promoted.^29^

## Kawasaki-like syndrome

The emergence in children of a Kawasaki-like disease strongly suspected of being associated with SARS-CoV-2 infection, points towards multiple pathogenic factors in COVID-19. As no unifying infection has been found in Kawasaki disease (KD), a multiple-agent model has been proposed for it. Unlike most infections, however, there are significant differences in ethnic predilection, which suggests a strong genetic influence. Various observations support this hypothesis: (i) KD prevalence is higher in East Asian populations, even in transmigration areas; (ii) African American children are disproportionately affected by KD as compared to Caucasian people^41^; (iii) nearly all instances of children with COVID-19-associated KD-like symptoms have occurred in western countries in which individuals of African descent are largely prevalent. In addition, Lo^42^ and Onouchi^43^ have described potential KD genetic susceptibilities such as *FCGR2A, BLK, CD40, ITPKC*, and *CASP3* from genome-wide association studies. Among the other factors implicated in the disease, Yoshida *et al*.^44^ reported that spontaneous NET formation was enhanced in the neutrophils of patients with acute KD, which would support our demonstration that COVID-19 is associated with NETs. As indicated above, host factors, in addition to comorbidities, appear to have a determining impact on COVID-19 severity. Individuals younger than 30 years old with no comorbidity have died from COVID-19, and only a low fraction of SARS-COVID-19 infected subjects are critically ill. We previously correlated the process of NETs formation with the genetic susceptibilities to KD described above.^45^ Overall, we speculate that host factors and/or genetic susceptibility are implicated in COVID-19, and hypothesize that these are associated with the innate immune response, in particular with the dysregulation of NETs and their by-products.^45^

## COVID-19 sequelae

The World Health Organization has said that the median time from COVID-19 onset to recovery is around 2 weeks for mild cases, and from 3 to 6 weeks for severe or critical forms of the disease. Many mild cases, however, have reported symptoms lasting far longer than 14 days. Symptoms that linger long after the initial infection have been observed, such as fatigue, muscle ache, rashes, breathlessness, muscle wasting, olfactory agnosia, slight neuropathies, and heart issues. While this might be explained by those patients’ stay in critical care units, a small fraction of diagnosed patients with mild symptoms stayed at home, yet nonetheless experienced serious illness sequelae after recovery and in viremia absence. At this time, no good data exist regarding long-term sequelae from COVID-19. While researchers are only now beginning to track the long-term health of survivors, past epidemics caused by other pathogens, such as Salmonella Typhi or SARS-CoV-2-type viruses, show that the aftermath can last more than a decade (i.e. 2003 SARS-CoV).

It is clear that these lingering symptoms are not directly caused by the virus, but are either the consequences of the body's recovery or of its inflammatory response, especially from systemic coagulopathies. The high incidence of thromboembolic events shown in autopsy findings^18^ suggests an important role of COVID-19-induced coagulopathy, which might result from the vicious cycle of auto-stimulation of NETs formation, resulting in an autoimmune-like disease.^28^ We speculate here that the dysregulation of NETs from SARS-CoV-2 infection is the principal event causing disease aftermath, and believe that such aftermaths might be reduced by testing for the progression of or predisposition to NETopathies.

## Environmental exposure

The high rate of SARS-CoV-2 transmission has generated considerable scrutiny. The contribution of aerosol transmission has now been established, as has that of droplets.^11^ It is therefore critical to mitigate viral shedding and inhalation exposure. This requires inward and outward mask protection, of between 0.02 to 1 µM and 0.2 to 1 µM, respectively.^11^ Monitoring airborne levels of SARS-CoV-2, as a way of identifying exposure hotspots, may be an alternative method for alerting people at increased risk of infection. This is particularly crucial in enclosed locations. Environmental exposure might also explain disparity in COVID-19 severity; individuals, or indeed populations around the globe, may not all be equal with respect to aerosol or fine particle susceptibility.

The virus can become airborne by attaching to the secretions (fine particles, nasal/saliva droplets) of an infected person, or to fine particulates suspended in the air. The latter are present in high concentration in atmospheric pollutants.

Highly populated or industrialized locations around the world have correlated with high virus transmission, allowing one to suggest atmospheric pollutants and in particular fine particles (principally produced from coil electric plants and vehicle traffic) as among the most obvious SARS-COVID-19 transmission factors. Fine particles might associate with viral particles directly, or may indirectly improve cell entry in host airways by their chronic inflammation of epithelial cell walls. It should also be noted that KD pathogenesis appears to be associated with certain environmental factors,^42^ as well as with a certain genetic background, and with infectious trigger pathogen-associated molecular patterns (PAMPs) or damage-associated molecular patterns (DAMPs). Besides seasonal factors, tropospheric wind patterns have also been linked to KD. It has been speculated that winds may carry fungal species or other environmental toxins that could trigger KD, and we may hypothesize that such conditions would favor chronic inflammation, stimulating formation of extracellular traps.

## Summary

Beyond a precision medicine for only detecting infected patients,^46^ this is to our knowledge the first article describing elements towards precision medicine for patients with COVID-19. Comorbidities, environmental and inheritance/genetic factors may all influence COVID-19 susceptibility. COVID-19 is a complex disease involving viral characteristics, and both innate and adapted immune responses, and as a consequence a high number of putative effectors. Identifying the reasons for these inequalities may reduce them. It may be possible to identify a panel of genetic alterations that are decisive in COVID-19 pathogenesis, especially those resulting in NETs dysregulation. The recognition of the varied and multiple factors that contribute to COVID-19 susceptibility and subsequent pathogenesis advocates the use of precision medicine in better designing clinical trials and in treatment of the disease.
